# Collective action within an environment of unknown unknowns: Experiences with the port of Mars Game

**DOI:** 10.1371/journal.pone.0308363

**Published:** 2024-08-05

**Authors:** Marco A. Janssen, Raksha Balakrishna, Lance Gharavi, Yi-Chun Hong, Allen Lee, Christine Nguyen, Michael Simeone

**Affiliations:** 1 School of Sustainability, Arizona State University, Tempe, Arizona, United States of America; 2 School of Music, Dance and Theatre, Arizona State University, Tempe, Arizona, United States of America; 3 Mary Lou Fulton Teachers College, Arizona State University, Tempe, Arizona, United States of America; 4 School of Complex Adaptive Systems, Arizona State University, Tempe, Arizona, United States of America; Ariel University, ISRAEL

## Abstract

There is limited research about how groups solve collective action problems in uncertain environments, especially if groups are confronted with unknown unknowns. We aim to develop a more comprehensive view of the characteristics that allow both groups and individuals to navigate such issues more effectively. In this article, we present the results of a new online experiment where individuals make decisions of whether to contribute to the group or pursue self-interest in an environment with high uncertainty, including unknown unknowns. The behavioral game, Port of Mars is framed as a first-generation habitat on Mars where participants have to make decisions on how much to invest in the shared infrastructure to maintain system health and how much to invest in personal goals. Participants can chat during the game, and take surveys before and after the game in order to measure personality attributes and observations from the game. Initial results suggest that a higher average social value orientation and more communication are the key factors that explain why some groups are more successful than others in surviving Port of Mars. Neither other attributes of players nor the group’s communication content explain the observed differences between groups.

## Introduction

Many of the challenges facing contemporary society can be categorized as collective action problems. Examples include emission reductions to reduce risks of climate change, vaccination for infectious diseases to create herd immunity, and the creation of community-driven knowledge systems such as Wikipedia or open-source software. In collective action, there is often tension or conflict between the goals of the community and the goals of the individual. Reduction of CO_2_ emissions is beneficial for current and future populations of this planet but bears costs to the individual. Wearing masks and hand washing during the COVID-19 crisis reduced the spread of the virus, although it might have been an inconvenience for the individual.

Governance of shared resources such as land, forests, water, fish stocks, or even knowledge are collective action problems. [1] coined the phrase “the tragedy of the commons” claiming that people cannot successfully govern their shared resources without private property regimes or governmental regulations. Ostrom [2] and her colleagues demonstrated that self-governance by communities is possible and defined a set of common design principles confirmed by empirical research using case studies and laboratory experiments. Nevertheless, there are still open questions [3].

Collective action is often studied empirically via the use of controlled experiments to advance causal knowledge [4]. In controlled experiments, researchers can manipulate specific contextual attributes of collective tasks that groups experience, which enables them to systematically test various hypotheses. These experiments are typically simple, abstract tasks with monetary tradeoffs for decision-making. Past experimental work has demonstrated that groups who are allowed to communicate in a common pool resource game without enforcing their promises (“cheap talk”) are effective in improving the level of cooperation [5,6]. Experimental research has also addressed collective action within the scope of environmental uncertainty and ambiguity [7,8]. What is not explicitly addressed is collective action with unknown unknowns.

Uncertainty can be approached in different ways. A common approach is to provide probabilities of specific events, which provide risks to the participants. Another approach describes the specific events, but probabilities are not provided. This kind of event is called ambiguous. As we will discuss in the next section, risk and ambiguity typically reduce the level of cooperation in common pool resource dilemmas. A third approach of uncertainty does not specify events; they are true surprises, also called “unknown unknowns” or “black swans” [9,10]. Given the unprecedented nature of global environmental change and the uncertainty we experienced during the COVID-19 pandemic, human society should continue to expect surprises that will impact, possibly catastrophically, sustainable futures. As such, experimental studies of how communities cope with the unexpected will be vital to the continued well-being of human communities, and to our species as a whole. Our experiment features novel ways to incorporate unknown unknowns.

The simple, abstract tasks in typical experiments in the social sciences, have sofar not explicitly included unknown unknowns. If uncertainty is addressed, it is the risk or ambiguity approach, and appears as a variation of probabilities within the payoff structure in the game. In our study, we merge practices from controlled experiments with serious games to study collective action in a more complex environment in which we explicitly include unknown unknowns.

In the next section, we discuss the theoretical background of the research questions. Then we discuss our experimental game Port of Mars, and present the results of a series of experiments. We close this paper with conclusions and implications for future research.

## Background

It is now well established that most people show some cooperative behavior in social dilemma situations where there is tension between individual and collective interests [11]. In fact, the human species is remarkably cooperative, not only with kin, but also with strangers, and utilizes sophisticated mechanisms of indirect reciprocity, such as gossip and costly signaling, to identify reputation and trustworthiness of strangers and to guide norms and social interactions [12,13].

Although humans are a cooperative species, there is a large diversity of outcomes of collective action among communities. For example, more than half of open-source software groups fail, meaning they never move beyond the initial stage of a project [14]. The current climate crisis, plastics in the oceans, air pollution in cities, and the depletion of groundwater around the world, are just a few of the many familiar examples of unsuccessful governance of the commons. In this study, we look at attributes of collective action problems that impact the ability of groups to successfully manage their shared resources.

### Uncertainty and surprises

Traditional experiments use dice or other randomizers to introduce uncertainty in rewards. With such methods, the probabilities and size of rewards are clearly indicated to participants. These experiments examine how participants deal with risk [7,8]. A common way uncertainty as risk is included in collective action experiments is with a probability of an increase or decrease in resource production, for example, due to extreme weather events. There are various studies on external shocks for collective action, all indicating a reduction of cooperation [7,15–17] except [18], which found that uncertainty increases cooperation when two groups interact in a social dilemma.

Another approach is the use of thresholds. Thresholds can be used to indicate a minimum amount of investment for a public good to be created [19], or a maximum amount of extraction from the common pool resource before it collapses [20]. When threshold levels are not known, we see a reduction in the level of cooperation with under-provision [21] and over-extraction [22,23]. When the probabilities of possible thresholds are known (risk treatment), some studies find that groups tend to have better outcomes than groups with completely uncertain thresholds (ambiguity treatment) [24], while others do not find a significant effect [25].

We can also consider thresholds that could be reached after various rounds instead of a one-shot situation, as happens in problems like climate change. [26] found that in such dynamic situations, a clearly communicated target to avoid a high probability of failure increases cooperation compared to lower probabilities of failure or not reaching the threshold. In field experiments with communication, uncertainty about thresholds in a dynamic resource game had only modest effects on increasing cooperation [26–28].

In all these experiments, the events that can happen are known to the participants. But what if the nature of the events is not known? We will discuss in the experimental design how we include unknown unknowns in our experiment.

### The effectiveness of communication

In a controlled commons dilemma experiment with no ability to enforce promises between participants, communication between participants should theoretically have no impact on participant outcomes if all participants are self-interested and rational decision-makers. However, allowing participants to communicate with one another has a major positive effect on average [29,30]. Researchers have posited several theories to explain this—it allows coordination among participants, it develops trust relationships, it expresses social pressure, etc.—but there is currently no conclusive explanation of why communication in commons dilemma experiments is effective. In this study, we look at the nature of communication between successful and unsuccessful groups.

Port of Mars is a game-based experimental platform we developed to investigate how unknown unknowns and communication impact performance in groups navigating social dilemmas. Port of Mars was originally developed as a project of Arizona State University’s Interplanetary Initiative to research how to maintain healthy human communities in space. It began as a physical card game about life on the first human community on Mars [31]. Our initial experience with Port of Mars as a research tool was promising since participants were deeply invested in the game due to its compelling narrative and how it demands players cope with deep uncertainty. Players didn’t know what could happen next: they were regularly faced with unknown unknowns.

Using Port of Mars as a behavioral game to study collective action under uncertainty, we address and test several specific hypotheses. In the game groups could succeed in surviving an unknown number of rounds, or fail. Since we aim to understand why some groups are better able to cope with unknown unknowns, we distinguish between groups that survived and those groups that failed. We test several attributes of participants and their interactions (communication) that can explain those differences. As such, we test the following hypotheses:

1. Surviving groups consist of participants who are more pro-social [32].
2. Surviving groups consist of participants who are more risk averse [33]
3. Surviving groups consist of participants who give more consideration to future outcomes [34]
4. Individuals who win the game act more selfishly [31]
5. Surviving groups communicate more [6]
6. Surviving groups have a more constructive tone of communication [30]

To increase accessibility and standardization of data collection, we developed a web-based multiplayer version of the game. Port of Mars is different from typical social dilemma experiments in its use of narrative, as opposed to monetary remuneration, to motivate subject performance. Although fishery framing and narratives have been used in early commons experiments [35–37], we use it strategically in Port of Mars. The project team includes artists and game designers to craft an engaging and compelling narrative that motivates players to emotionally invest in the stakes of the game. Using narratives also allows us to explicitly include unknown unknowns in our experiments.

Using the web-based version of Port of Mars, we ran a “Mars Madness” tournament where participants could play multiple full games, depending on their in-group performance, and compete with one another to become the Port of Mars champion. For a participant to succeed, the groups they participate in need to work together to solve collective action problems. However, participants also have incentives to free ride on the actions of others. We used the “Mars Madness” tournament as a vehicle to attract participation, but in our present analysis we only use data from Round 1 where all participants were new to the game.

The fictional Martian community of Port of Mars provides a helpful context for examining sustainability. Spaceships have been used as metaphors for sustainable use of shared resources [38]. If one has limited space and resources to share, and alternatives are not realistically available, sustainable use of the shared resources is needed to survive. This “spaceship economics” approach is contrasted with “cowboy economics” approach often observed on Earth. The latter is characterized by extraction, depletion, and moving on to new locations [38]. The hazardous Martian environment leaves very little room for errors in resource management. With the current enthusiasm for space exploration among the broader public, our use of a Martian context is appealing and provides a compelling context in which to investigate the set of urgent and analogous challenges that confront humanity today.

Space exploration is hard. Getting to Mars and surviving there will require overcoming a broad set of technological and engineering challenges. Yet the social aspects of sending humans to Mars, or elsewhere in the solar system, may prove the greatest challenge of all. Inhabitants of the Red Planet will rely on one another for their well-being and survival. How can people most effectively navigate dilemmas of shared resources, and collective action in the context of high uncertainty?

## Methods

Port of Mars is a resource allocation game where five players must balance individual goals and achievements against the conflicting needs of maintaining shared infrastructure called System Health, in the face of ongoing environmental, social, and technical challenges. The narrative identifies the players as members of Generation Zero: the first group of long-term residents to arrive on the Red Planet, and the Players experience the challenges of life as early citizens of a Martian settlement. To survive, they must navigate between their personal ambitions and the group’s needs.

**Fig 1:**
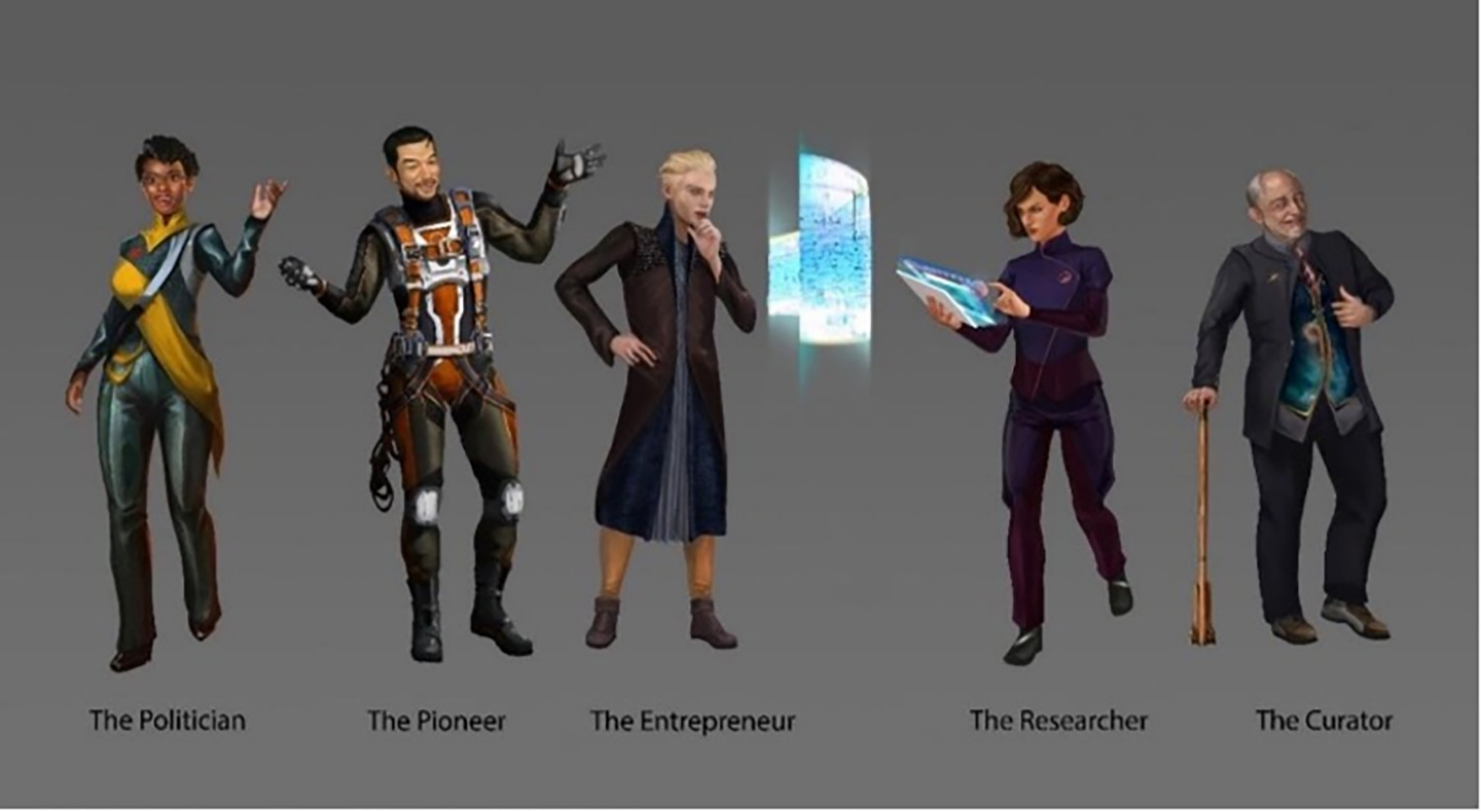
Illustrations of the five characters in the card game of Port of Mars (source: [31]).

There are five roles in the game that are uniquely assigned to each individual player: the Curator, Pioneer, Researcher, Politician, and Entrepreneur (Fig 1). Although the roles have a different narrative, the underlying payoff structure is the same for each role. This is information not known by the players. Shared infrastructure degrades due to wear and tear and external events in the game. Players must maintain a minimum level of shared infrastructure for the game to continue and the community to survive. At the same time, they are each individually trying to win the game. Players may spend their personal resources either on maintaining the community or on acquiring points needed to win. As such, the game experience provides a fundamental social dilemma where there are tensions between individual goals and collective goals.

In the game, “System Health” is a number that represents the physical health of the community—the condition of infrastructure, production of food, water, and breathable air, radiation shielding, energy production, and other necessary resources. This number begins at 100 at the start of the game, and goes down by 25 points each round, representing wear and tear on infrastructure and consumption of resources. If System Health reaches zero, the community does not survive, and everyone dies. No one wins if everyone is dead.

Each player receives ten “Time Blocks” per round. These blocks can be spent to improve System Health or to pursue their own ambitions. Each Time Block spent on System Health raises the System Health number by one point. Players may also spend Time Blocks to purchase Influence cards. These cards represent the player’s capacity to impact specific domains. There are five kinds of Influence cards: Culture, Legacy, Science, Governance, and Finance. Players can spend combinations of their accrued Influence cards to purchase Accomplishment cards, which represent significant achievements in their domain—e.g., scientific discoveries, cultural productions, political milestones, etc., and allow players to earn points to win the game.

Each player may earn one type of Influence Card cheaply, and two other types at greater expense. Each player has two types of Influence cards they may *not* earn through investment of Time Blocks. For example: the Politician may exchange two Time Blocks for one Governance card, three Time Blocks for a Culture or Legacy card, but may *not* exchange their Time Blocks for Science or Finance cards. To acquire Influence cards, they are unable to earn directly, players may arrange trades with one another. Under normal circumstances (barring certain Events which are described in more detail below), players can communicate with each other through a group chat window that is always available during the game. Once players have the right combination of Influence cards, they may use them to purchase an Accomplishment card that will give them points. At the end of the game, if the community survives, the player with the most points wins.

Each round also features unexpected developments in the form of random Event cards. These Events can lead to reductions of System Health (e.g., dust storms, solar flares, crop failures) or other consequences, positive or negative. Normally, players draw one Event card per round. If System Health falls below 65, they draw two Event cards, and if it falls below 35, players draw three Event cards. With more than 10 events there are millions of possible sequences of events. Each game is unique, and the possibility of events creates uncertainty, especially since players do not know what kind of events are possible.

Players must thus choose how to allocate their resources each round. Their decisions are secret and hidden from the other players. Do they pursue only their own goals and free-ride on the resources of the community? Or do they contribute to the good of the community and its shared resources? The players can chat with each other during the whole game, and any chat message will be visible for anyone else. We will analyze the content of the chat messages later in the manuscript to evaluate how it relates to outcomes of the game.

In order to recruit participants, we created Mars Madness Tournaments at our university during Fall 2021 and Spring 2022 (October 15, 2021 –March 25, 2022). The tournament was open to all undergraduate students from the university. High-performing players of surviving groups, those who got more points than the majority of players in the group, were invited to participate in subsequent tournament rounds until we had one remaining group of participants who played for the championship. We organized this tournament as a way to attract participants. Winners of the tournament would earn a prize of $1000 USD, and the players at the championship game level all received a Port of Mars t-shirt. Participants were recruited via email invitations, social media, and messages from instructors. This research was approved by our university’s Institutional Review Board and research ethics committee.

Before and after the game, we held brief surveys to collect information about attributes of the participants (see https://osf.io/zr76e/ for details). Since we are interested in how players participate in collective action in a complex dynamic environment, we wanted to collect information about social value orientation, willingness to take risks, and ability to plan and remain patient to wait for rewards. Those are common metrics to evaluate individual attributes of players, and we use this to evaluate whether individual attributes can explain the performance of individuals and groups in the experiments [32]. We asked a direct question on whether they consider themselves to be on a scale from 0 to 10 “a person that is fully prepared to take risks or a person to try to avoid taking risk”, which has been found to be a robust way to collect information about risk preference [39,40]. We used the 6-question slider measurement of [41] to measure social value orientation, wherein for each question, the person is asked to select a distribution of resources distribution to self vs another. The appendix of [41] details how those six questions are used to calculate the SVO of the person on the spectrum of altruistic to competitive. The outcome of the SVO measurement is an angle. If this angle is above 57.15° the person is identified as altruistic, if the angle is between 22.45° and 57.15° the person is identified as pro-social, if the angle is between -12.04° and 22.45° the person is identified as individualistic, and if the angle is smaller than -12.04° the person is identified as competitive. This SVO metric is found to have the strongest correlation with decisions in social dilemma [32]. A14-question instrument of [42] is used to measure players’ consideration of the consequences for the future of their decisions. The value of the future thinking measurement is between 14 and 98. Will those who are eager for immediate gratification be less likely to invest in system health? To measure patience, we included the time discount measurement from [43]. The participant is asked their preference in a series of questions for an immediate reward versus a higher reward in the future, identifying impatient (score = 1) up to very patient (score = 32) individuals. In the post-game survey, the participants were asked which player they identified as the leader of the group.

The team chose to analyze texts using straightforward statistical methods found in corpus linguistics. To analyze the text extracted from the chat-based conversations of game sessions, we divided the text logs into “not survived” and “survived” datasets, creating text corpora for each. For comparison of the two corpora, keywords were calculated using a log-likelihood measure to calculate which words appeared more frequently in one corpus than another when allowing for the total population of terms in each corpus. The keywords for the ‘survived’ corpus were listed in order of frequency, creating a list of terms distinctive to that corpus when compared to the “not survived” corpus. The same list was created for the “not survived” corpus as compared to the “survived” text. Top terms from each list were then examined using a Keywords in Context (KWIC) examination, and a collocate analysis [44] was performed as well for a handful of terms at the top of each list to gain better perspective on their usage on context in each corpus.

In the next section, we discuss the findings from Round 1 of the Mars Madness tournaments in Fall 2021 and Spring 2022. The participants were all students at Arizona State University. In Round 1 of each tournament, participants could log in during two daily launch time windows for a period of five to seven days. Participants had to provide informed consent to sign up and participate in this experiment, complete a survey, and watch an instructional video before they could enter a virtual lobby to join a game. Once at least five participants were present in the lobby, connected participants would be randomly assigned to groups of 5, and individual games with each group of 5 would begin. The virtual lobby closed 30 minutes after the scheduled launch time, and players who were unable to be assigned to a game could try another time. Each player could only participate in a single game in Round 1.

## Results

**Fig 2:**
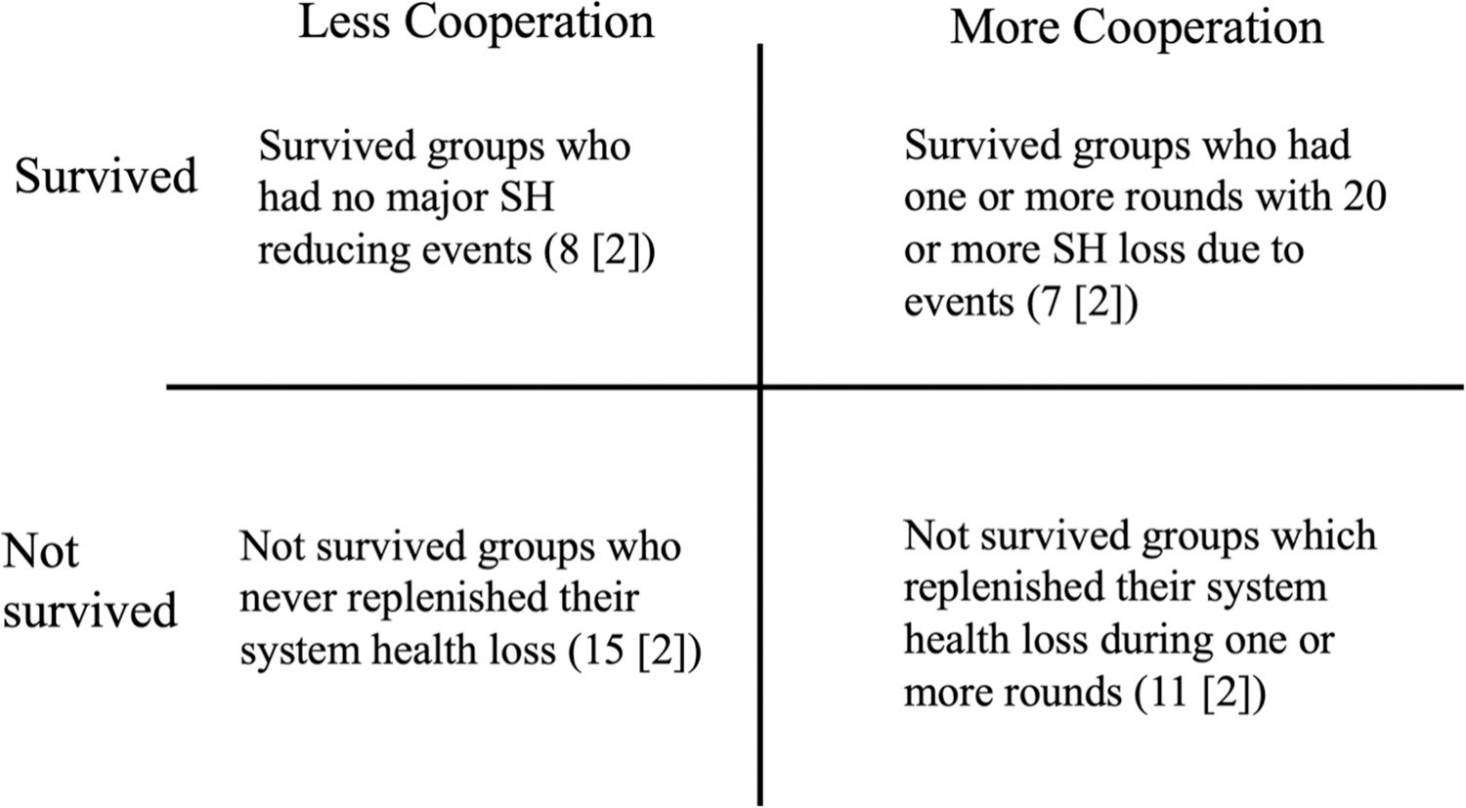
Typology of games played. Between parentheses, we see the number of games, and between brackets, the number of games where one or more bots were present. SH refers to System Health.

**Fig 3:**
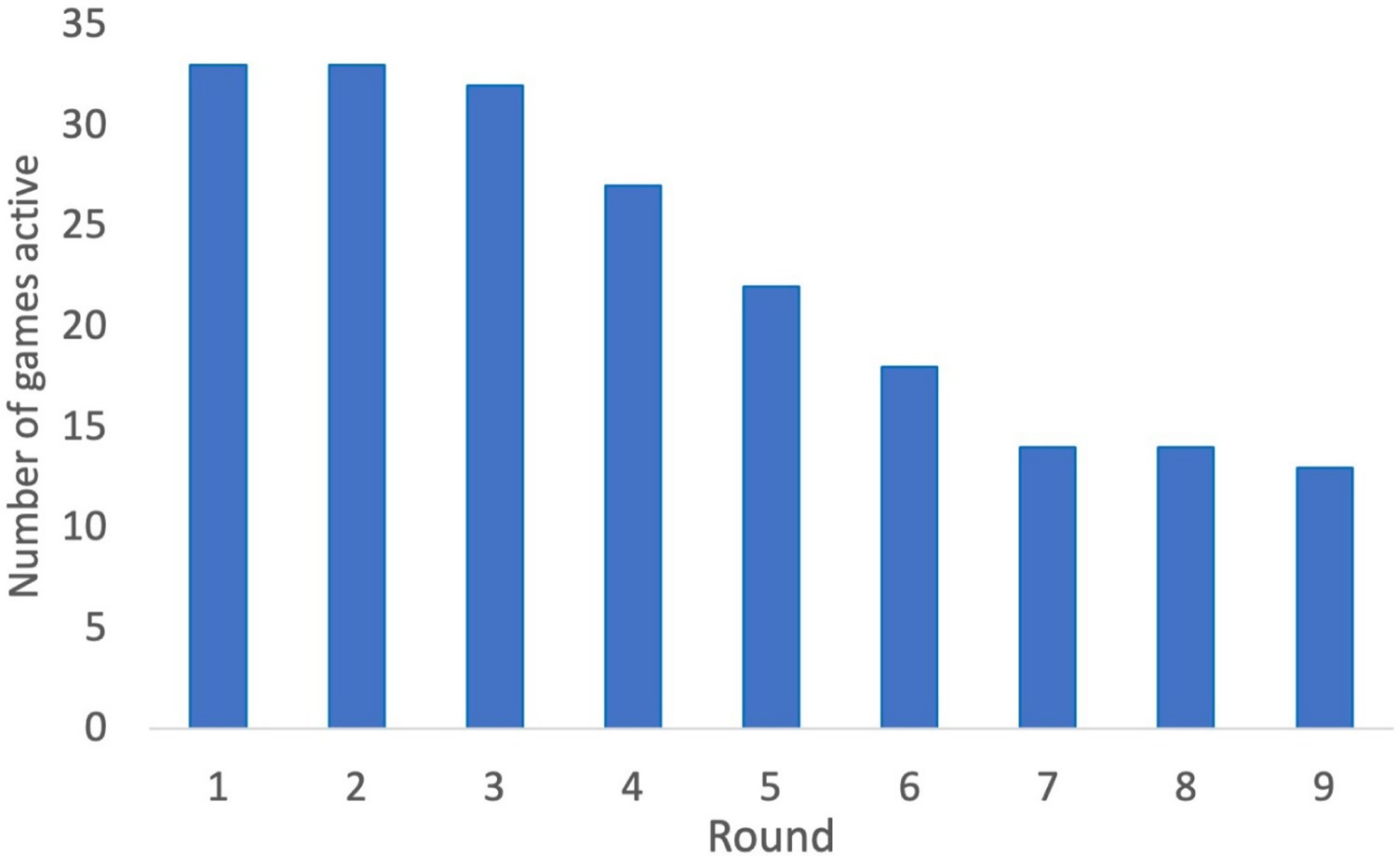
The number of games active at the start of the round.

**Fig 4:**
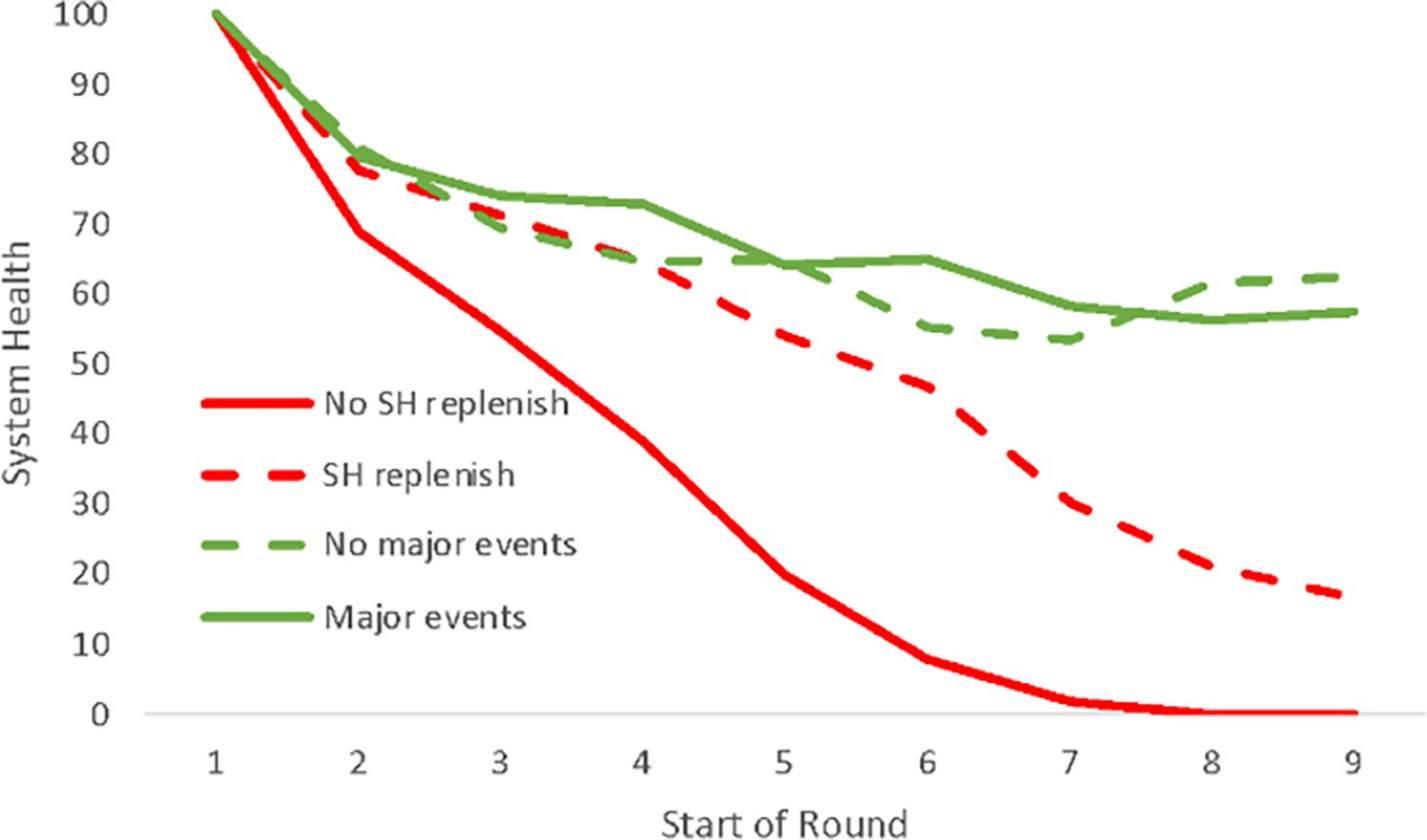
The average system health level at the start of the round for active groups for the 4 categories (only including the 33 groups without bots).

A total of 41 games with 205 participants were played. Of the 41 games, 15 survived till the end, and 26 did not survive. There was a lot of variability among the games. Some games ended within 5 rounds, while others survived despite major events reducing their system health. We identified 4 types of gameplay (Figs 2–4). Among the survivors, there were 7 groups who experienced one or more rounds with major system health-reducing events and were still able to survive—a strong indicator of high cooperation. Eight groups did not experience these kinds of major events, so less cooperation was needed to survive. Among the groups that did not survive there were 15 groups who never reinvested the amount of system health lost due to wear and tear and external events during any round. As such, their system health declined systematically to zero. In 11 other groups who did not survive, there was at least 1 round in which they reinvested their system health loss. Data for the 41 games, including the survey instruments, are available at https://osf.io/zr76e/.

As is not uncommon in online experiments, there were players who dropped out [45]. If a player was disconnected in Port of Mars or did not perform any in-game actions for 5 minutes, their player was taken over by a bot that would perform default actions for them. This happened to 11 of the 205 players. Bots accept any trade request for which they have sufficient Influence cards to trade, always attempt to earn at least 2 of their specialty Influence card while investing all remaining time blocks into System Health, and purchase any Accomplishment cards they are able to. They do not communicate. Bots replaced players in 8 of the 41 games, and because this could impact the outcome of the groups, we removed those games from the data set for further analysis. The appearance of bots was equally spread across the four different categories shown below. For the remaining 33 groups and 165 players, we investigated whether there were differences in player actions, survey responses, and communication across these categories.

We tested the difference in participants in surviving and non-surviving groups by using a Mann–Whitney–Wilcoxon rank sum test, a non-parametric test to test whether two samples are likely from the same distribution, and found no significant effect on risk-preference, time-discounting, future thinking, and gender (Table 1). 45% of group members of surviving groups are male, while 57% of group members of non-surviving groups are male. However, individuals from groups who survived had a significantly (p<0.05) higher level of pro-social values compared to those who did not survive. Of the 55 individuals from surviving groups, 48 were identified as prosocial, 6 as individualistic, and 1 as competitive. Of the 110 individuals from groups that did not survive, 88 were identified as prosocial, 21 as individualistic, and 1 as competitive. This provides support for hypothesis 1 (pro-social), but not for hypotheses 2 (risk-aversion) and 3 (future thinking).

**Table 1:** Mean values of individual attributes from participants in groups that survived and not survived. The p-value indicates whether there is a significant difference between the two types of groups, which is only the case for social value orientation.

Attribute (range)	Group survived	Group did not survive	p-value
Male	45%	57%	0.153
Social value orientation (-20,75)	32.60	29.12	0.013
Risk aversion [0–10s]	5.95	6.07	0.452
Future thinking [14,98]	70.80	68.23	0.211
Patience [1,32]	18.05	17.44	0.784
N	55	110	

From the 11 groups that survived, what explains which players won? To find out, we performed a multi-level logit regression where the dependent variable is whether a player won in their group (1) or not (0). The independent variables are individual measurements of the value of social-value orientation, future thinking, whether the individual identifies as male (= 1) or not, the patience and risk preference score, the share of communication, investments and trade during the first 3 rounds of the game (the start of the game, which is experienced by all groups). We recognize that there might be an effect of 5 individuals in the same group, which is why we use multi-level regressions with the group as an additional level. Our regression analysis shows that the multi-level analysis had a significant effect (prob > chi2 in Table 2). Table 2 shows that future thinking and a self-centered orientation are important personality attributes for victorious players, supporting hypothesis 4 (selfish). We also found that winning individuals contribute relatively more to chat communication at the start of the game (hypothesis 5 supported).

**Table 2:** Results from a multi-level logit regression analysis on the likelihood of winning the game, where we list the direction and significance of the effects for 4 variations of factors.

	Model 1	Model 2	Model 3	Model 4
Pro-social values	- *	- **	- **	- **
Future thinking	+ **	+ *	+	
Male	+			
Patience	-			
Risk preference	+			
Share communication (first 3 rounds)			+ **	+ **
Share investments (first 3 rounds)			-	
Share trade (first 3 rounds)			+	
N	55	55	55	55
prob > chi2	0.0437	0.0220	0.0211	0.0058
Pseudo R^2	0.21	0.14	0.24	0.19

*/**/*** stands for a significant effect with respectively p<0.1, p<0.05 and p<0.01.

Using a multi-level linear regression for all 1095 decisions on how much to invest in System Health, we find that participants’ decisions relate to personality attributes and context of the current game. (Table 3). We removed 12 decisions when an Event card forced a player to invest all their time blocks in System Health. The dependent variable is the level of investment, and the independent variables are system health (and its quadratic expression), the round of the game, the maximum level that can be invested (capacity which can be changed due to event cards), the cost of events (measured in reduction of system health as stated on the event cards), playing a screw card this or last round (where screw card is a card that gives the player points at the cost of system health reduction of the group), fraction of chat messages, whether the person identifies as male, social value orientation, risk preferences, patience, future thinking and whether the event card “difficult decisions” is present, which doubles the costs of investments. Since individuals in groups may impact each other, we use multi-level regression, with an additional level for the group.

**Table 3:** Results from multi-level linear regressions to explain investment levels of individuals for each round.

Predictors	Predicted Investment	Predicted Investment (Group survived)	Predicted Investment (Group not survived)
Constant	-1.663 (1.276)	-0.306 (2.023)	-3.188 (1.592)*
System Health	0.142 (0.031)***	0.035 (0.049)	0.182 (0.036)***
System Health ^ 2	-0.0013 (0.0002)***	-0.001 (0.0003)*	-0.002 (0.0003)***
Round	0.128 (0.039)***	0.115 (0.043)**	-0.034 (0.088)
Capacity	0.128 (0.017)***	0.590 (0.052)***	0.077 (0.017)***
Event costs	0.069 (0.015)***	0.077 (0.017)***	0.046 (0.028)
Plays Screw card	0.085 (0.027)**	0.062 (0.53)	0.100 (0.030)***
Played Screw card last round	0.041 (0.030)	0.116 (0.054)**	0.024 (0.035)
Others Played Screw cards last round	-0.003 (0.015)	-0.008 (0.029)	0.006 (0.017)
Fraction chat messages	1.012 (0.511)*	1.554 (0.700)*	0.755 (0.674)
Gender (Male = 1)	-0.361 (0.184)*	-0.423 (0.259)	-0.300 (0.240)
SVO	0.017 (0.007)**	0.015 (0.009)	0.015 (0.009)
Risk Preference	-0.079 (0.049)	-0.105 (0.068)	-0.007 (0.065)
Patience	0.004 (0.008)	0.012 (0.010)	-0.0033 (0.010)
Future Thinking	0.014 (0.007)	0.001 (0.010)	0.024 (0.010)*
Difficult Decisions	-2.238 (0.297)***	-2.081 (0.414)***	-1.870 (0.389)***
N	1083	547	536
Number of groups	33	11	22
-Log Likelihood	2566.804	1266.804	1241.706
Variance components			
Individual level	0.581 (0.235)	0.533 (0.309)	0.453 (0.280)
Group level	6.437 (0.283)	5.796 (0.355)	5.771 (0.365)
*χ* ^2^	24.46 (p<0.001)	15.97 (p<0.001)	6.71 (p<0.01)

* p<0.05

** p< 0.01

*** p<0.001.

From the individual attributes of the player, a higher social value orientation (more cooperative) leads to more investment into System Health. There was also a modest gender effect where male players invested less in System Health. Risk preference, patience, and future thinking had no significant impact. Again, this supports hypothesis 1 (pro-social), but not hypotheses 2 (risk aversion) and 3 (future thinking). If Event cards reduce System Health, participants invest more. If Event cards reduce the number of Time Blocks available for a player (capacity), they invest less. There is a quadratic relationship between investments and System Health. A System Health around 50 leads to higher investment levels than higher or lower levels of System Health. If System Health is high, one could afford to invest less in System Health without major impacts. If System Health is very low, this is a sign that a low-performing group may have given up investing in the shared infrastructure. The fact that “round” has a positive relationship is caused by the fact that groups must cooperate to invest in the shared infrastructure to survive for many rounds.

There are two Accomplishment cards that give a player various points but cost System Health. When a player plays one of these cards, that player receives points at no cost to them personally, but at an often steep cost to the community in the form of reduced System Health. The players called them “screw cards,” and we saw that players who used those cards invested more in System Health to compensate for the lost System Health, but not enough to recover the total reduction in System Health caused by their use of the “screw card.”

Another context of the game that can impact System Health investments is the volume of chat messages. Those who contributed more to the communication volume invested more in the System Health, which provides support for hypothesis 5.

There are a few differences between groups that survived and those that did not. Groups that did not survive, did not increase investments when big Events happened, and so did not increase investments over time.

From the 47 individuals from surviving groups who identified a leader in their group, 18 out of the 47 times these identified leaders won the game. Hence winners are more likely to be identified as leaders of the group.

**Fig 5:**
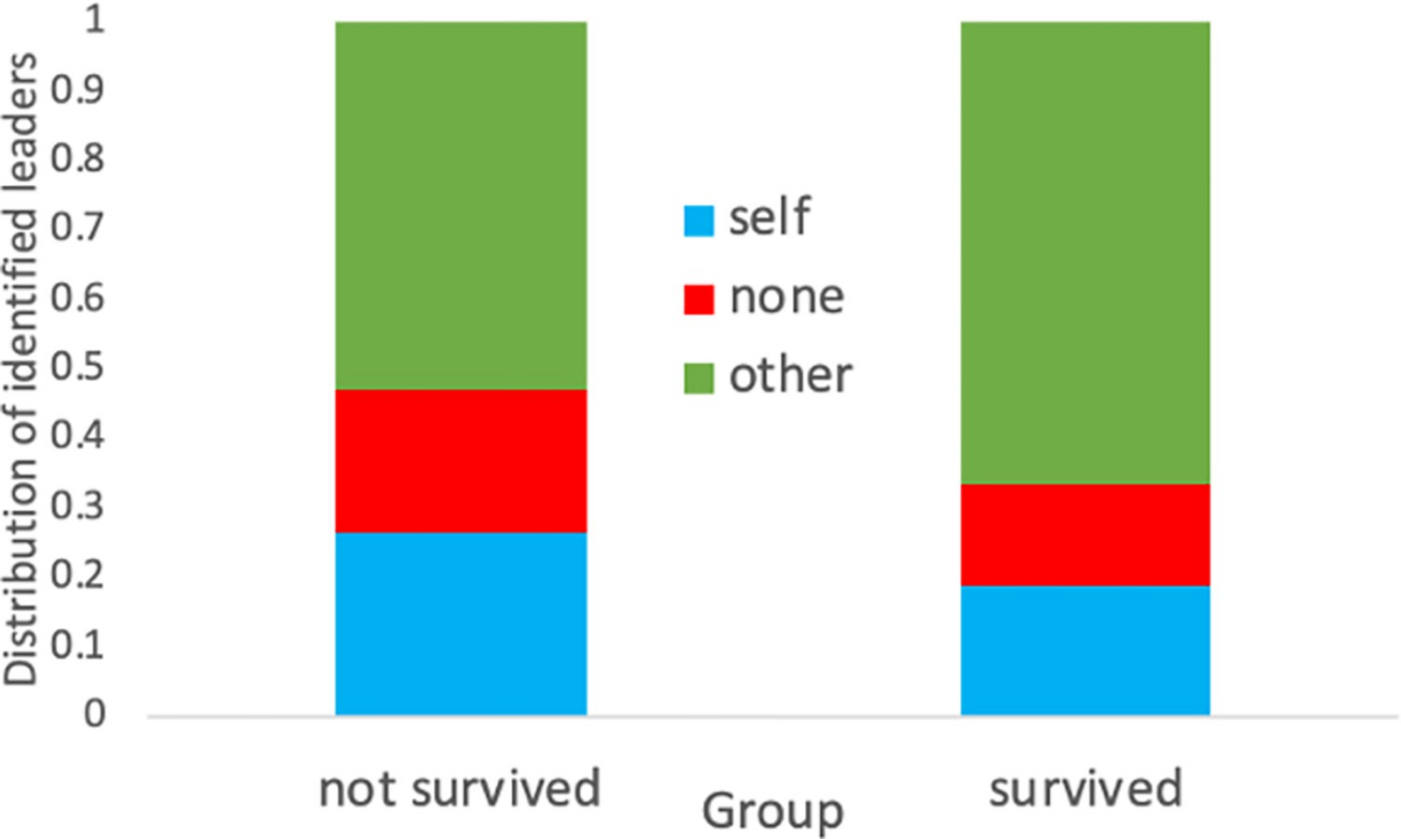
Distribution of which players are identified as leaders.

If we look at all groups, we see a difference in the individuals who are identified as leaders (Fig 5). Individuals from groups who did not survive are more likely to either identify themselves or nobody as a leader. Individuals from groups who survived identified other members (67%) as leaders compared to (53%) of groups who did not survive. Perhaps, successful groups are more likely to value others who stood up to lead them.

**Fig 6:**
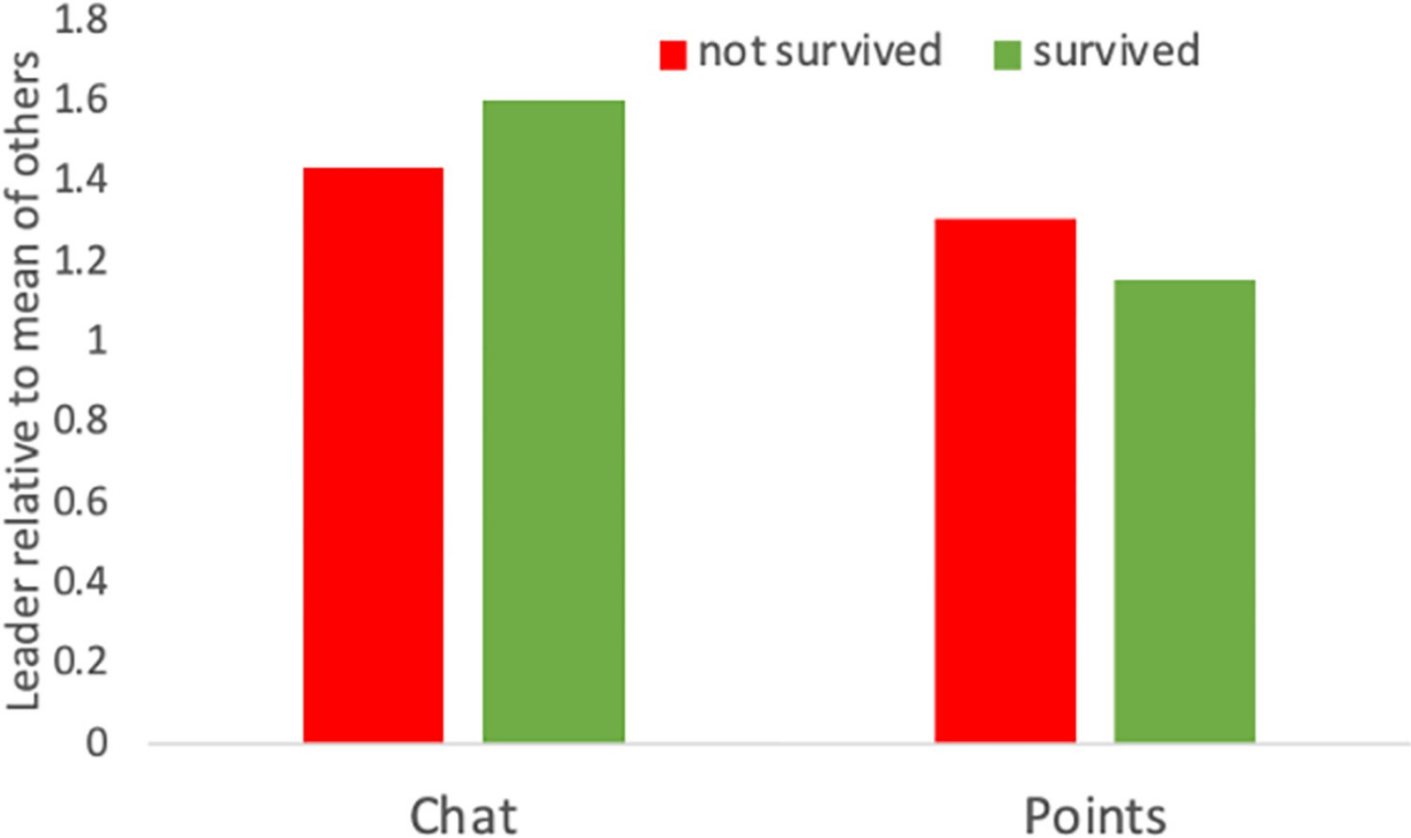
Relative chat messages and points of leaders vs others in groups that survived or not.

Whether groups survived or not, individuals identified as leaders communicated more than the mean of others in the group. They also earned more points (Fig 6). We recognize that people can have different concepts of leadership when asked to identify the leader of the group. But in line with the card game version of the Port of Mars game [31], leaders are considered to lead communication and do well in the game.

From analysis of the in-game communication, we see that there is a difference in the amount of chat/communication within groups that survived and those that did not survive. On average, groups that survived communicated 10.5% more than groups that didn’t survive (based on unigram term frequency and normalized per round), which support hypothesis 5 (communication). The difference in the amount of communication is similar between the two most distant group categories “survive/more cooperation” and “not survive/less cooperation”– 10.1%. The difference in the usage of ‘we’ and ‘I’ between groups that survived and those that not survived is .5% and 1% respectively and not statistically significant. Between the group categories “survived/more cooperation” and “not survive/less cooperation groups.” terms such as *vote*, *health*, *I*, *all*, *hero*, *everyone* are used more by the “survived/more cooperation group.” but that does not directly indicate a different type of communication.

Almost 48% of the communication was initiated by players identified as leaders and/or winning individuals. These players seemed to steer the conversation and took initiative in setting common goals–such as investment in System Health. Words such as *remember*, *maintenance*, *critical*, *maxed*, *planning*, and *contribute* are the most used words with *system* and *health*, indicating their role in leading discussions on managing the system. The following are some illustrative examples:

“There are 5 of us, if we all do 7, that’s only 35, let’s all do 8?”

“Guys if we play the super long game and only buy 1 thing each round, and use the rest on system health it will eventually guarantee us a win; Are y’all down with that?”

“We could also think about a turn hiatus where everyone invests all points into health”

They also talk significantly about losing in a way of cautioning against letting system health fail.

“Oof, we need health; we have to survive; or we all lose”

“We have to invest all of the blocks in health; or else we won’t make it”

In some instances, they also seem to encourage the team, and appreciate efforts to improve system health.

“Ay, good job team”

“THERE WE GO EVERYBODY”

From the chat, it is evident that they took initiative during the game, proposed game strategies, planned for investment in System Health and encouraged group decisions to avoid failure in the game.

Groups that survived communicated 32.2% more than groups that not survived during events or disturbances. This indicates that groups that survived communicate more with each other when faced with uncertain events when compared to groups that don’t survive. We measure volume of communication by unigram count. This indicates that the amount of chat initiated by groups that survived, during rounds with one or more events leading to a reduction of System Health with 10 or more units is much higher than that of the groups that not survived. *Hero* and *pariah* seem to be the distinct terms characteristic of these situations when compared to other rounds, but these only indicate game technical terms.

[30] found that communication in highly cooperative groups was more constructive. However, sentiment analysis of the Port of Mars communication did not identify a difference in sentiment between survived and not-survived groups. Hence, we do not find support for hypothesis 6 (constructive tone).

## Discussion

In this study we presented initial results from the web-based Port of Mars game, an online multiplayer game that focuses on collective action and uncertainty. We find that the only personality attribute that explains performance of groups and individuals is social value orientation. This is the most effective indicator found in [32] to explain cooperation in social dilemmas. We found that the average social value orientation in surviving groups is more prosocial than those of groups who did not survive (Hypothesis 1 confirmed). We found that individuals who won the game were more self-interested than others in the group (Hypothesis 4 confirmed). Other personality attributes, such as risk-aversion, future thinking, and time-discounting, did not explain outcomes (Hypotheses 2 and 3 rejected). We also found that groups that survived exchanged more chat messages per round (Hypothesis 5 confirmed), and that leaders identified by the players themselves communicated more than the average of other players. However, quantitative analysis of communication content did not reveal significant differences (Hypothesis 6 rejected).

We did not find individual or group attributes that provide insight into why some groups cope better with unknown unknowns than others. There is always uncertainty about the actions of other players, social uncertainty. Since communication was allowed, some of this uncertainty can be mitigated. The Port of Mars game is unique in explicitly including surprises. We did not find explanations for why some groups are able to cope with surprises better than others. Individual attributes such as risk aversion and future thinking did not provide strong correlations, and the communication content did not reveal clear patterns either. A larger sample of groups, and additional survey instruments with different treatments might be needed to understand the variations.

## Conclusions

How do groups cope with collective action in environments with unknown unknowns? We find that social-value orientation, a personality trait commonly found to correlate with high levels of cooperation, also provides an explanation for successful groups in this study. Furthermore, more communication is found to correlate with successful groups, although we could not find a significant difference in the content of the communication.

Given the complexity of the game and the diversity of gameplay, the small sample of 33 groups is a limitation of this study. Especially post-pandemic, it is increasingly a challenge to recruit individuals to participate in group experiments, which hinders data collection. Additional analysis of the communication patterns is underway to study the role of leadership and leadership styles. Such an analysis of the qualitative data enables us to derive a better understanding of the role of leaders and the group dynamics and how they differ between groups that survived and those that did not.

We only had one treatment and did not compare the game’s results if there were no unknown unknowns. Future studies could include different levels of variability, for example, varying the number of cards of “Life as Usual” to derive treatments with differences in the number of surprises they experience.

Another future treatment to consider are possible interventions that increase the probability of groups to survive. A possible intervention is the use of prompts to advise groups to invest more into system health if they drop below certain levels of system health.

In all, the initial findings presented on experiments with the Port of Mars game provide insights into the ability of groups to cooperate even when exposed to unknown unknowns. As commonly found in social dilemma research, a higher average social value orientation is the key factor for groups to succeed in the challenge to survive the challenges on the fictional Mars habitat.
